# Schockraumauslastung in deutschen Traumazentren: Versorgungsrealität von O&U in Reform unterrepräsentiert

**DOI:** 10.1007/s00113-025-01538-1

**Published:** 2025-02-19

**Authors:** Cora R. Schindler, Florian Pavlu, Philipp Faul, Philipp Störmann, Dan Bieler, Uwe Schweigkofler

**Affiliations:** 1 Klinik für Unfallchirurgie und Orthopädie, Goethe Universität, Frankfurt am Main, Theodor-Stern-Kai 7, 60590 Frankfurt am Main, Deutschland; 2 Klinik für Unfallchirurgie und Orthopädie, Berufsgenossenschaftliche Unfallklinik Frankfurt/Main, Frankfurt am Main, Deutschland; 3 Klinik für Unfallchirurgie und Orthopädie, Wiederherstellungs- und Handchirurgie, Verbrennungsmedizin, Bundeswehrzentralkrankenhaus Koblenz, Koblenz, Deutschland; 4 Klinik für Orthopädie und Unfallchirurgie, Universitätsklinikum Düsseldorf, Medizinische Fakultät, Heinrich-Heine-Universität Düsseldorf, Düsseldorf, Deutschland

**Keywords:** Gesundheitspolitik, Notfallversorgung, Schockraumalarmierung, Polytrauma, Vorhaltung, Health policy, Emergency care, Emergency room, Trauma-team-activation, Infrastructure provision

## Abstract

**Einleitung:**

Der Gemeinsame Bundesausschuss (G-BA) veröffentlichte im April 2018 eine Regelung zu einem gestuften System von Notfallstrukturen an Krankenhäusern (nach Sozialgesetzbuch V) und legte damit die Grundsätze für Notfallversorgung im stationären Sektor fest. Die Strukturanforderungen für die Versorgung von potenziell Schwerverletzten wird primär durch die Fachgesellschaft definiert und bedeutet eine hohe personelle Vorhaltung bei gleichzeitig jährlich zunehmender Auslastung bei steigenden Patientenzahlen. Ziel der Studie ist es, die tatsächliche Auslastung der traumatologischen Schockräume in zertifizierten Traumazentren zu erfassen.

**Methodik:**

Hierzu wurde eine deutschlandweite Online-Umfrage unter 619 zertifizierten Traumazentren DGU® durchgeführt, um die Häufigkeit der Schockraumalarmierungen zu quantifizieren. Die Ergebnisse wurden retrospektiv mit den Daten aus den Jahresberichten 2021 und 2022 des TraumaRegister DGU® verglichen.

**Ergebnisse:**

Die beteiligten Kliniken meldeten im Jahr 2021 22.479 und im Jahr 2022 24.366 Schockraumalarmierungen (Rücklaufquote der Online-Antworten von 24,1 %). Von diesen Alarmierungen entfielen 70 % auf überregionale Traumazentren. Die Vergleichsanalyse zeigte, dass die realen Fallzahlen 3‑ bis 5‑mal höher sind als die im TR-DGU® dokumentierten Daten. 80 % der Schockräume wurden aufgrund geringer Verletzungsschwere nicht im Register erfasst.

**Zusammenfassung:**

Die Studie belegt die Diskrepanz zwischen der dokumentierten und der tatsächlichen Auslastung der Schockräume in deutschen Traumazentren und betont die Notwendigkeit, die finanzielle und personelle Berücksichtigung der Traumazentren an die reale Inanspruchnahme anzupassen, um eine weiterhin hohe Versorgungsqualität zu gewährleisten.

**Graphic abstract:**

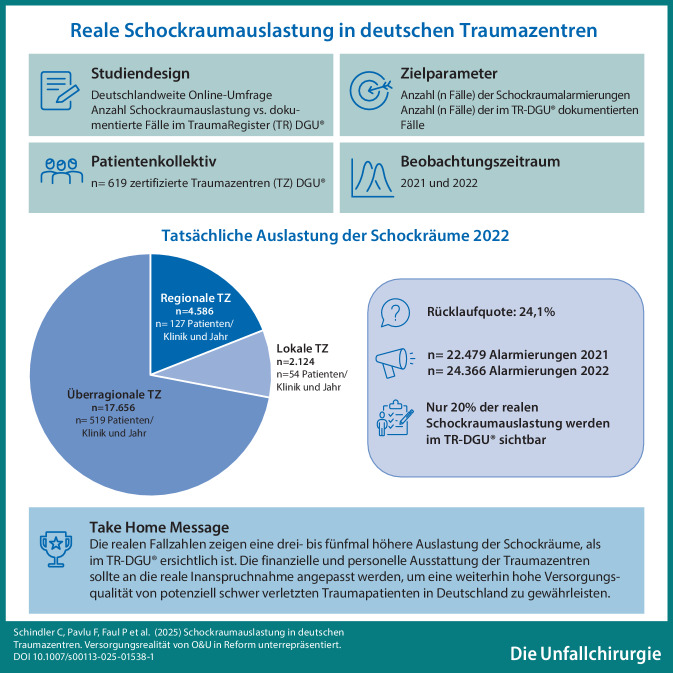

**Zusatzmaterial online:**

In der Online-Version dieses Beitrags (10.1007/s00113-025-01538-1) finden Sie den in der Studie verwendeten Fragebogen.

## Abstract (en)

**Introduction:**

The Federal Joint Committee (G-BA) published a regulation on a staged system for emergency structures in hospitals (according to Social Security Code V) in April 2018 and therefore established the foundations for emergency care in the inpatient sector. The structural requirements for the care of potentially severely injured patients is primarily defined by the specialist society and means a high provision of personnel with a simultaneous annual increase in the burden due to increasing numbers of patients. Critics fear that the reforms do not take sufficient account of trauma care both professionally and financially. The aim of the study was to record the actual workload of the emergency trauma rooms in certified German trauma centers.

**Method:**

A nationwide online survey was performed in Germany among 619 trauma centers certified by the German Society for Trauma Surgery (DGU®) to quantify the frequency of trauma room callouts. The results were retrospectively compared with the data from the annual reports of the TraumaRegister (TR) DGU® from 2021 and 2022.

**Results:**

The 167 participating hospitals reported 22,479 trauma room callouts in 2021 and 24,366 in 2022 (response rate of the online survey 24.1%). Of these callouts 70% concerned level 1 supraregional trauma centers. The comparative analysis showed that the actual number of callouts is 3–5 times higher than the data documented in the TR-DGU® . Of the trauma rooms 80% were not included in the register due to low injury severity.

**Summary:**

The study confirms the discrepancy between the documented and actual workload of the trauma rooms in German trauma centers and emphasizes the necessity to adjust the financial and personnel consideration of the trauma centers to the actual utilization in order to ensure a continuing high quality of care.

## Einleitung

Der Gemeinsame Bundesausschuss (Oberstes Beschlussgremium der gesetzlichen Krankenversicherung in Deutschland, G-BA) hat am 19.04.2018 eine Umstrukturierung der innerklinischen Notfallversorgung beschlossen. Die Reform löste in der Fachgesellschaft Sorgen um die zukünftige Versorgungsqualität von Patienten mit Verletzungen und muskuloskeletalen Krankheitsbildern aus. Kritiker fürchten eine fachliche wie finanzielle Unterrepräsentation von Orthopädie und Unfallchirurgie in der innerklinischen Notfallversorgung [16].

Der Beschluss sieht nach Art und Umfang der Notfallvorhaltungen 3 Versorgungsstufen vor (Basisnotfallversorgung – Stufe I, erweiterte Notfallversorgung – Stufe II, umfassende Notfallversorgung – Stufe III). Für jede Stufe sind Mindestvoraussetzungen bezüglich Personal und technischer Ausstattung, die mit entsprechenden finanziellen Zuschlägen bedacht werden, definiert. Notfälle sollen überwiegend in einer eigenständigen, fachlich unabhängigen Zentralen Notaufnahme (ZNA) versorgt werden [10]. Bereits die *Basisnotfallversorgung *soll über eine Fachabteilung Chirurgie/Unfallchirurgie sowie einen Schockraum (SR) verfügen.

Ein Krankenhaus wird der *erweiterten Notfallversorgung* zugeordnet, sofern es ein spezialisiertes Krankenhaus, das die Anforderungen eines überregionalen Traumazentrums gemäß *Weißbuch Schwerverletztenversorgung* erfüllt und 24/7 an der Notfallversorgung teilnimmt, ist [5, 10].

Im Jahr 2021 wurden 9,8 Mio. ambulante Notfälle in deutschen Krankenhäusern behandelt, davon liegt bei etwa 50 % der Fälle ein unfallchirurgisches bzw. orthopädisches Krankheitsbild vor [3, 22]. Der traumatologische Schockraum ist dabei hochfrequentiert, was die permanente Verfügbarkeit eines Facharztes aus O&U erfordert. In den Jahren 2005–2009 lag das Verhältnis von Schockraumpatienten, dokumentiert im TR-DGU®, zur Anzahl schwer verletzter Patienten (ISS ≥ 16) bei ca. 2:1. Marzi et al. zeigten eine kontinuierliche Zunahme der Schockraumzuweisungen von 70 % über 10 Jahre auf ein Verhältnis von > 3:1. Diese Entwicklung bedingt einen erhöhten Vorhaltebedarf personeller wie struktureller Ressourcen sowie finanzielle Mehrbelastung für die Traumazentren [13, 18].

Das TraumaRegister® der Deutschen Gesellschaft für Unfallchirurgie (TR-DGU®) liefert als multizentrische Datenbank epidemiologische und strukturelle Daten zur (Schwer)Verletztenversorgung in Deutschland. Die wissenschaftlich-fachliche Leitung des Registers obliegt der Sektion Notfall‑, Intensivmedizin und Schwerverletztenversorgung (NIS) der DGU. Sie setzt sich u. a. für Maßnahmen der Qualitätssicherung in der Traumaversorgung ein. Jährlich werden über 38.000 Fälle aus knapp 700 Kliniken dokumentiert [12]. Daten über die tatsächliche Inanspruchnahme der traumatologischen Schockräume in Deutschland fehlen allerdings. Denn bei Weitem nicht alle behandelten Patienten erfüllen nach Abschluss der Schockraumbehandlung die Einschlusskriterien wie eine entsprechende Verletzungsschwere bzw. intensivmedizinische Behandlung [2]. Aufgrund des hohen Aufwands für diese hochspezialisierte Behandlung ist aus unserer Sicht eine Erhebung der Gesamtzahl der SR-Alarmierungen als Diskussionsgrundlage der realen Belastung der Unfallchirurgie und der eingebundenen Fachdisziplinen essenziell. Denn selbst bei einer SR-Alarmierung ohne aufwendige Behandlung sind die Ressourcen vorzuhalten. Ziel der Studie ist es, die tatsächliche Auslastung der traumatologischen Schockräume in zertifizierten Traumazentren zu erfassen. Wir möchten aufzuzeigen, dass nur ein Teil der geleisteten Arbeit/des Ressourceneinsatzes dokumentiert wird und daher nicht in Diskussionen herangezogen werden kann.

## Methodik

Die vorliegende Studie soll die tatsächliche Inanspruchnahme der traumatologischen Schockraumversorgungen ermitteln. Zur Quantifizierung der Schockraumalarmierungen wurde mit dem Arbeitskreis Umsetzung TraumaNetzwerk DGU® (AKUT) eine deutschlandweite Umfrage durchgeführt. Am 27.11.2023 wurde die *Questionstar*-Online-Umfrage nur an die jeweiligen ChefärztInnen oder StandortleiterInnen der zertifizierten TraumaZentren DGU® (TZ-DGU®, *n* = 619) versandt, um Doppelantworten auszuschließen. Gefragt wurde nach der tatsächlichen Häufigkeit der SR-Alarmierungen in den Jahren 2021 und 2022. Außerdem wurde gefragt, wie viele Fälle im TR-DGU® dokumentiert bzw. aus welchen Gründen nicht dokumentiert wurden.

Die Vergleichsdaten stammen aus den Jahresberichten 2021 und 2022 des TR-DGU® [11, 12]. Diese beinhalten eine retrospektive Auswertung prospektiv gesammelter, pseudonymisierter Patienten- und Alarmierungsdaten. Das in den Jahresberichten beschriebene Basiskollektiv umfasst Patienten, die über den Schockraum aufgenommen und anschließend entweder einer Intensivtherapie zugeführt werden oder vorher versterben. Zusätzlich muss mindestens ein Maximaler-Abbreviated-Injury-Scale(MAIS)- Schweregrad ≥ 3 vorliegen bzw. MAIS 2 in Kombination mit Versterben oder einer Aufnahme auf Intensivstation (ITS) [2, 9].

## Ergebnisse

### Umfrageergebnisse der beteiligten TraumaZentren-DGU®

Die Rücklaufquote der Umfrage betrug 24,1 % (Deadline 30.01.2024). Insgesamt beteiligten sich *n* = 167 Kliniken an der Umfrage, davon waren *n* = 53 lokale, *n* = 61 regionale und *n* = 53 überregionale Traumazentren.

In den an der Umfrage teilnehmenden Kliniken (Tab. 1) wurden die Behandlungsteams 22.479-mal (2021) bzw. 24.366-mal (2022) durch Schockraumalarmierungen beansprucht. 70 % der Fälle entfielen auf ÜTZ, 20 % auf RTZ und 10 % auf die LTZ. Die durchschnittliche Schockraumauslastung der ÜTZ lag damit bei *n*_*∅*_ = 494 Patienten/Klinik und Jahr (RTZ: *n*_*∅*_ = 122 Patienten/Klinik und Jahr ; LTZ *n*_*∅*_ = 53 Patienten/Klinik und Jahr ).

**Tab. 1:** Auswertung der Umfrage

	2021			2022		
	LTZ	RTZ	ÜTZ	LTZ	RTZ	ÜTZ
*Anzahl gültiger Rückmeldungen (n)*	38	37	35	38	37	35
*Schockraumalarmierungen (n)*	1926	4158	16.395	2124	4586	17.656
Anteil am Gesamtkollektiv (%)	9	18	73	9	19	72
Patienten/Klinik und Jahr	51	116	468	54	127	519
*n* als MW	40	90	327	39	100	325
*n* als Median (IQR)	(17–70)	(60–120)	(200–604)	(18–92)	(60–140)	(199–715)
*Eingabe TR-DGU® (n)*	510	1209	4248	522	1140	3987
Keine Eingabe TR-DGU® (*n*)	1416	2949	12.147	1981	3446	13.669
Fehlende Einwilligung (%)	10	9	14	9	9	16
Andere Gründe (%)	7	4	10	7	3	10

*n* als MW und Median (*IQR)*; Die tatsächliche Auslastung der Schockräume lag damit bei 40/39 (2021/2022) Patienten/Klinik und Jahr in den LTZ. Die Umfrage ergab *n* = 90/100 Patienten/Klinik und Jahr in den RTZ. Die ÜTZ gaben eine Auslastung von *n* = 327/325 Patienten/Klinik und Jahr an. Die Verteilung des Gesamtkollektivs zwischen den Traumastufen war ca. 10 % LTZ, 20 % RTZ und 70 % ÜTZ

Nach Ergebnissen der Umfrage wurden in den Jahren 2021 und 2022 in den LTZ *n*_*∅*_ = 516 Fälle im TR-DGU® dokumentiert. In den RTZ wurden *n*_*∅*_ = 1175 und durch die ÜTZ *n*_*∅*_ = 4118 Fälle eingegeben. LTZ gaben an, *n*_*∅*_ = 149 Patienten aufgrund fehlender Datenschutzeinwilligung nach DSGVO nicht in das TR-DGU® eingeschlossen zu haben. In den RTZ fehlten *n*_*∅*_ = 275 und in den ÜTZ *n*_*∅*_ = 1972 Einwilligungen. LTZ gaben an, *n*_*∅*_ = 110 Fällen aufgrund anderer, z. B. organisatorischer Gründe nicht dokumentiert zu haben. In den RTZ waren es *n*_*∅*_ = 114 Schockräume, in den ÜTZ *n*_*∅*_ = 1276.

### Versorgungsrealität vs. Registerdaten

Tab. 2 gibt einen Überblick über die im TR-DGU® dokumentierten Fälle für die Jahre 2021 und 2022 [11, 12]. Daraus ergeben sich durchschnittlich *n*_*∅*_ = 40 Patienten (*n*_*∅*_ = Durchschnittswert) pro TZ-DGU® und Jahr. Innerhalb des Basiskollektivs sind 55 % der Patienten schwer verletzt mit einem Injury Severity Score (ISS) ≥ 16 [17]. Über 30 % erleiden lebensgefährliche Verletzungen [14] und 15 % (2022) ein Polytrauma gemäß „Berlin Definition“ [15]. Über 80 % der Verletzten werden intensivmedizinisch behandelt.

**Tab. 2:** Dokumentierte Schockraumbehandlungen aus den Jahresberichten TR-DGU® 2021–2022

	2021	2022
*Dokumentierte Patienten gesamt (n)*	35.747	38.544
*Nichtbasiskollektiv (n)*	7167	7738
*Basiskollektiv (n)*	28.580	30.806
Injury Severity Score (ISS) ≥ 16 (*n*)	15.424 (54 %)	16.866 (55 %)
Lebensgefährlich (schwer) Verletzte* (*n*)	8804 (31 %)	9707 (32 %)
Polytrauma** (*n*)	4006 (14 %)	4591 (15 %)
Intensivbehandlung (*n*)	23.903 (84 %)	25.894 (84 %)

Die gezeigten Daten wurden den veröffentlichten Jahresberichten 2021 und 2022 des TR-DGU® entnommen [9, 11]

Abbreviated Injury Scale (AIS): Jeder Verletzung wird ein Schweregrad zwischen 1 (leicht) und 6 (maximal) zuordnet. Daraus werden Schweregradangaben wie der „Maximale AIS-Schweregrad“ (MAIS) oder der „Injury Severity Score“ (ISS) berechnet. Basiskollektiv: Alle Patienten mit MAIS ≥ 3; Patienten mit MAIS 2, die entweder verstorben sind oder auf einer Intensivstation waren

* ISS ≥ 16 kombiniert mit physiologischen Traumafolgen wie bei der Polytrauma-Definition (Paffrath et al. [17], Pape et al. [16]). ** „Berlin Definition“: mindestens 2 Körperregionen relevant verletzt, und es liegt mindestens ein physiologisches Problem vor [16]

60 % der Patienten werden in ÜTZ versorgt, etwa 30 % in regionalen (RTZ) und 10 % in lokalen Traumazentren (LTZ). Die durchschnittliche Verletzungsschwere der Patienten in den LTZ liegt bei einem ISS 14, in den RTZ bei ISS 16 und in den ÜTZ bei ISS 20. Über 80 % der Patienten werden im primär aufnehmenden TZ-DGU® weiterbehandelt. Knapp 20 % der LTZ und ca. 10 % der RTZ verlegen ihre Verletzten innerhalb von 48 h nach der Aufnahme. 11 % der Patienten werden den ÜTZ sekundär zuverlegt (Tab. 3).

**Tab. 3:** Patientenkollektiv nach Versorgungslevel LTZ, RTZ und ÜTZ aus den Jahresberichten TR-DGU® 2021–2022

	2021			2022		
	LTZ	RTZ	ÜTZ	LTZ	RTZ	ÜTZ
Anzahl der Kliniken (*n*)	297	232	130	290	225	133
Anteil am Gesamtkollektiv (%)	11	30	59	11	29	60
Patienten/Jahr und Klinik (n*)	10	35	123	10	36	124
Injury Severity Score (ISS)	13	16	20	14	16	20
ISS ≥ 16 (%)	33	46	60	34	46	61
Lebensgefährlich (schwer) Verletzte* (%)	7	11	18	7	10	18
Polytrauma** (%)	17	26	36	17	26	36
SHT, AIS_Kopf_ ≥ 3 (%)	17	26	42	19	28	43

n* wird im Jahresbericht in MW angegeben

*SHT* Schädel-Hirn-Trauma, *AIS* Abbreviated Injury Scale [12]

* Verletzungsschwere von ISS ≥ 16 ist kombiniert mit physiologischen Traumafolgen wie bei der Polytrauma-Definition (Paffrath et al. [17], Pape et al. 2014, [16]). ** Gemäß der „Berlin Definition“ sind mindestens 2 Körperregionen relevant verletzt, und es liegt mindestens ein physiologisches Problem vor (Pape et al. 2014, [16]). Schweres Schädel-Hirn-Trauma (SHT) = AIS_Kopf_ ≥ 3 [12]

Überträgt man die Umfrageergebnisse auf die Anzahl der deutschlandweit zertifizierter TZ-DGU® (2021 *n* = 659, 2022 *n* = 648), lässt sich die reale Schockraumnutzung hochrechnen. Daraus ergeben sich Auslastungen von rund 65.000 Patienten in den ÜTZ, 25.000 in den RTZ und 15.000 in den LTZ pro Jahr. Damit werden in Deutschland, extrapoliert, über 100.000 Patienten jährlich in den zertifizierten traumatologischen Schockräumen versorgt. Im Vergleich dazu wurden in den Jahren 2021 und 2022 *n*_*∅*_ = 37.146 Fälle im TR-DGU® dokumentiert; das entspricht 34 % der tatsächlichen Inanspruchnahme. Vergleicht man die mittleren Fallzahlen (Fälle/Klinik und Jahr) im TR-DGU® mit den Werten der Umfrage, liegt die tatsächliche SR-Auslastung in den LTZ 5‑mal (*n*_*∅*_ = 10 vs. 53), in den RTZ 3‑mal (*n*_*∅*_ = 36 vs. 122) und in den ÜTZ 4‑mal (*n*_*∅*_ = 124 vs. 494) höher als die Registerzahl. Bei hochgerechnet über 100.000 SR-Alarmierungen pro Jahr wären entsprechend unseren Ergebnissen ca. 70.000 Schockräume nicht erfasst. Laut TR-DGU® liegt der zeitlichen Versorgungsaufwand der Schockraumbehandlung bei mindestens 172.000 Arbeitsstunden/Jahr (Dauer: Aufnahme bis ITS = 103,7 min). Eine Vollzeitstelle entspricht etwa 1700 h/Jahr [19, 24]. Hochgerechnet entspricht der Arbeitsaufwand also 110 Vollzeitstellen – allein für das SR-Basisteam (mindestens 2 Pflegekräfte und 2 Ärzte) sind das 440 Vollzeitstellen.

## Diskussion

Im Jahr 2021 stellten sich rund 26.800 Menschen/Tag in deutschen Notaufnahmen vor [22]. Bieberthaler et al. haben über einen 6‑monatigen Zeitraum 393.587 ambulante Notfälle in der Stadt München gezählt. 43 % der Patienten entfielen auf das Fachgebiet von O&U [3]. In einem Gutachten der Deutschen Gesellschaft für Interdisziplinäre Notfall- und Akutmedizin e. V. (DGINA) handelte es sich bei 21 der 25 häufigsten Notfalldiagnosen um ein Krankheitsbild aus O&U, 6 % sind Arbeits‑, Wege- oder Schulunfälle. Unfallchirurgische Krankheitsbilder können aufgrund notwendiger Diagnostik wie konventioneller Bildgebung oder Computertomographie sowie der aufwendigen Versorgung komplexer Verletzungen häufig nicht außerhalb des Krankenhauses behandelt werden [7]. Die aufwendigste Primärbehandlung aus dem unfallchirurgischen Bereich entfällt dabei unstrittig auf potenziell schwer verletzte Patienten, die über den SR aufgenommen werden [24]. Das TraumaRegister DGU® liefert valide Daten, wie viele Patienten nach initialer Behandlung tatsächlich schwer verletzt waren. Zahlen über die reale Auslastung der Ressource Schockraum, inklusive der nach Abschluss der Behandlung als übertriagiert identifizierten Patienten, fehlen bislang. Anhand unserer Umfrage zeigen wir erstmals, dass die traumatologische SR-Auslastung mit hochgerechnet über 100.000 Alarmierungen bundesweit pro Jahr um das 3‑ bis 5Fache höher liegt als die im TR-DGU® dokumentierten Zahlen. Der Anteil der tatsächlich als schwer verletzt ins TR-DGU® aufgenommenen Patienten liegt bei ca. 34 % der abgelaufenen Schockbehandlungen. Diese sind mit hohen personellen und strukturellen Vorhaltungen der zertifizierten Traumazentren verbunden, um die Struktur- und Qualitätsanforderungen zu erfüllen.

Die Relevanz einer verfügbaren Unfallchirurgie wird im aktuellen G‑BA-Beschluss zur Reform eines gestuften Systems der Notfallversorgung sichtbar. Bereits die *Basisnotfallversorgung – Stufe I* soll einen Schockraum (traumatologisch und konservativ) mit 24-stündig verfügbarer CT bereitstellen. Ein Krankenhaus wird der Stufe II – *erweiterte Notfallversorgung* zugeordnet, sofern es die speziellen Anforderungen eines ÜTZ erfüllt [5, 10]. Laut Beschluss soll die ZNA als eigenständige, fachübergreifende Einheit fungieren. Die ärztliche Leitung soll über die Zusatzweiterbildung „Klinische Notfall- und Akutmedizin“ verfügen (S. 3 § 6, S. 4 § 9). Ein bundesweit einheitliches Leitungsmodell gibt es nicht. Grundsätzlich muss eine fachärztliche Behandlung binnen 30 min gewährleistet sein [10]. Für die Polytraumaversorgung gibt es durch die Fachgesellschaft, sowohl im Weißbuch als auch der S3-Leitlinie Schwerverletztenversorgung klar definierte strukturelle Voraussetzungen [5, 6]. Dementsprechend soll die SR-Versorgung in festen, interprofessionellen Teams nach vorstrukturierten Plänen erfolgen. Das Team soll ein spezielles Training absolviert haben (z. B. *Advanced Trauma Life Support®*) und die notfallmedizinische und -chirurgische Kompetenz abbilden, d. h. im Zweifelsfall auch notfallchirurgische Eingriffe vornehmen können [1, 5, 6]. Störmann und Marzi formulierten bereits auf dem Deutschen Kongress für Orthopädie und Unfallchirurgie 2023 die Erwartungen von O&U an die ZNA: Aufgrund der relevanten Patientenzahl mit Akutpathologien des Bewegungsapparates und der hohen Auslastung traumatologischer Schockräume kann auf eine fachliche Kompetenz aus O&U in der Notaufnahme nicht verzichtet werden [23]. Für die Schwerverletztenversorgung muss eine Umsetzung der definierten Standards von den Notaufnahmen eingefordert werden; diese wird mit der beschriebenen 30-min-Frist bis zum Eintreffen eines Facharztes nicht gewährleistet. Die Basisversorgungsstufe nach G‑BA setzt die Vorhaltung eines Schockraums voraus, daraus ergibt sich formell die Anwesenheit eines Facharztes für O&U in der Notaufnahme 24 h 365 Tage im Jahr. Ein nichtunerheblicher Teil der traumatologischen Schockräume sind Arbeits- und Wegeunfälle, die die Rahmenbedingungen für eine besondere Heilbehandlung erfüllen. Nichtdelegierbare Tätigkeiten sind u. a. die definitive Diagnosestellung, Entscheidung über und Durchführung der besonderen Heilbehandlung sowie invasiver Therapien. Hierfür ist die Verfügbarkeit einer fachunfallchirurgischen Expertise mit D‑Arzt-Qualifikation erforderlich [21].

Die Kliniken gaben an, ca. 20 % der Patienten wegen fehlender Einwilligung oder z. B. aus organisatorischen Gründen nicht im TR-DGU® dokumentiert zu haben. Damit wurden rund 80 % der fehlenden Fälle aufgrund der geringen Verletzungsschwere nicht im TR-DGU® erfasst und müssen formell als Übertriage gewertet werden. In der internationalen Literatur wird für die sichere Versorgung von Schwerverletzten eine Rate von 30–50 % übertriagierter Patienten als unvermeidbar antizipiert, um eine intolerable Untertriage zu vermeiden. Unsere Daten zeigen auf, dass die Ressource Schockraum aktuell überproportional häufig in Anspruch genommen wird und somit einen vermeidbar hohen Personalaufenthalt erzwingt. In der oben genannten Studie von Marzi et al. trugen insbesondere nichtarztbegleitete Patienten und SR-Alarmierungen nach Unfallhergang zur steigenden Inanspruchnahme bei. Die Zahl der tatsächlich Schwerverletzen blieb konstant [13]. Wichtig sind deshalb klare Empfehlung zu SR-Alarmierungskriterien [20]. Deshalb wurden die Indikationen für eine SR-Alarmierung in der aktuellen Version der S3-Leitlinie Schwerverletztenversorgung intensiv überarbeitet. Für festgelegte Verletzungsmuster, Maßnahmen und pathologische Befunde werden nach dem Grad der zugrunde liegenden Evidenz (Grade of Recommendation, GoR) GoR-A- und GoR-B-Kriterien unterschieden sowie eine praktikablere Nomenklatur eingeführt. Die GoR-A-Kriterien entsprechen in Teilen den Empfehlungen des American College of Surgeons zur „field triage“ (FT) in der I. und II. Stufe und werden als „high risk for severe injury“ eingeordnet (HRSI = GoR-A). Die GoR-B-Kriterien entsprechend der III. Stufe der FT, hier geht man von einem mittelschweren Risiko für schwere Verletzungen (MRSI = GoR-B) aus. Zur Schärfung der akkuraten Schockraumzuweisung wurden u. a. Kriterien, wie das ∆ 30 km/h, eliminiert [6]. Zu berücksichtigen ist jedoch, dass ein Drittel der Zuweisungen nicht aufgrund einer Leitlinienempfehlung erfolgt, sondern durch eine „provider decision“. Eine Studie der NIS zur Qualität der Schockraumzuweisungen zeigt die Problematik bzw. Schwierigkeit einer adäquaten prähospitalen Triage. Dies führt u. a. zu der hohen Diskrepanz zwischen SR-Alarmierungen und den im DGU-TR® abgebildeten Fällen [19].

Die Bereitstellung einer interdisziplinären Versorgung von Schwerverletzten ist personal- und ressourcenaufwendig [1, 20]. Modernste Räumlichkeiten (mindestens 25 m^2^), Diagnostikeinheiten (Sonographie, Computertomographie in räumlicher Nähe) sowie qualifiziertes Personal in ausreichender Kapazität sind 24/7 vorzuhalten. Das Virchow-Klinikum publizierte im Jahr 2000 jährliche Gesamtkosten von 24 Mio. DM für die Polytraumaversorgung, davon waren 7 Mio. DM reine Vorhaltekosten [8]. Schoppow et al. berechneten ein Kostendeckungsdefizit von 5858 €/Schwerverletztem durch das aG-DRG-System [18]. Die Studie von Verboket et al. stützt diese Aussage [24]. Der G‑BA-Beschluss sieht entsprechend der Versorgungsstufe gestaffelte Zuschläge für die Beteiligung an der Notfallversorgung vor. Krankenhäuser sollen jährlich für die *Basisnotfallversorgung* 153.000 €, 459.000 € für Stufe II und für die *umfassende Notfallversorgung* 688.500 € erhalten [10]. In einer Pressemitteilung kritisierte DGOU-Vorstandsmitglied und Fachbeirat der Deutschen Interdisziplinären Vereinigung für Intensiv- und Notfallmedizin (DIVI), Prof. Dr. Seekamp, die Finanzierung läge weit unter den Erwartungen und dem Bedarf von O&U. Die Notaufnahmen blieben mit ihren hohen Vorhaltekosten unterfinanziert [16]. In der Reform der Krankenhausvergütung wurden 3 Hauptversorgungsstufen erarbeitet, um lokale, regionale und überregionale Versorgungsaufträge abzugrenzen. Ein Krankenhaus der Grundversorgung (mit Notfallstufe I) hält mindestens die Fachabteilungen Innere Medizin und Chirurgie mit einer ärztlichen 24/7-Präsenz und einer definierten Notfallversorgung vor. In Zukunft sollen die Krankenhäuser nicht mehr pro Fall (aG-DRG-System) bezahlt werden, sondern dafür, dass sie im Notfall Patienten behandeln können [4]. Unsere Daten zeigen, dass bereits in der Grundversorgungsstufe (Level I) und *Basisnotfallversorgung* mit einer hohen Zahl an traumatologischen Schockräumen und, damit verbunden, hohen Vorhaltekosten für die fachgerechte Behandlung der Patienten gerechnet werden muss. Als übergeordnetes Ziel für die Zukunft ist sicherlich auch die Erlangung eines validen ICD-10-Codes mit entsprechender OPS der Schockraum- und Schwerverletztenversorgung, um in Zukunft gerechter über das DRG-System abgebildet zu sein.

### Limitierungen

Die Limitierungen der Studie liegen sicherlich in der Rücklaufquote der Online-Umfrage von 24 %. Sichtet man hierzu Zahlen aus der Literatur, wird ersichtlich, dass allgemein eine Rücklaufquoten > 20 als „zufriedenstellend“ bewertet wird (s. Survey Anyplace, Genroe). Weiterhin ist die unterschiedliche Handhabe zur Generierung der internen Zahlen nicht klar erfasst worden. Aus vielen Kliniken wissen wir, dass meistens ein händisches Schockraumbuch geführt wird. Die Art der Erhebung wurde aber im Fragebogen nicht erfasst. Hier ist sicherlich eine Subjektivität der Beobachter möglich. Eine Konsequenz unserer Umfrage war die Implementierung von Erhebungsbogen als Initiative der lokalen Traumazentren, welche in Zukunft besser dokumentativ abgebildet werden möchten. Ebenso ist die Extrapolation der Gesamtzahl der traumatologischen Schockräume nur als Schätzung anzusehen.

## Fazit für die Praxis

Unsere Auswertung belegt eine enorme Ressourcenbelastung und kann als quantitative Datengrundlage herangezogen werden, um die finanzielle und strukturelle Ausstattung der traumatologischen Schockraumversorgung bedarfsgerechter zu planen. Dieser personelle wie strukturelle Aufwand ist aus Sicht von O&U in der aktuellen Reform nicht ausreichend abgebildet. Es wird deutlich, dass ein Registergesetz zur Gewährleistung der Datenqualität unerlässlich ist. Hier sind nach Einführung der neuen HRSI- und MRSI-Alarmierungskriterien in der neuen S3-Leitlinie aus dem Jahr 2022 eine stringente Einhaltung und Schulung zu fordern. Die Fachgesellschaft spricht sich klar für die Teilhabe von O&U an der Notaufnahmenleitung und für mehr Träger der Zusatzbezeichnung „Klinische Akut- und Notfallmedizin“ in O&U aus.

## Supplementary Information

Hier finden Sie die Zusammenfassung zur Datenerhebung in den Kliniken.

## Data Availability

Die Daten, die die Ergebnisse dieser Studie stützen, sind auf begründete Anfrage und mit Genehmigung bei den Autoren erhältlich.
