# Supplementary material for: Trauma room workload in German trauma centers: Reality of trauma care underrepresented in reform
Source: Unfallchirurgie (Heidelb). 2025 Feb 19;128(6):434–40. [Article in German] doi: 10.1007/s00113-025-01538-1 (PMC12116947; doi:10.1007/s00113-025-01538-1)
Supplement: Supplementary file 1 — Hier finden Sie die Zusammenfassung zur Datenerhebung in den Kliniken. [file 113_2025_1538_MOESM1_ESM.pdf]

**Appendix**

Online-Fragebogen extrahiert:

Q1. Bitte geben Sie die Stufe Ihres TraumaZentrums an

Q2. Wie viele traumatologische Schockraum-Alarmierungen erfolgten in Ihrem Haus?

Bitte geben Sie die Anzahl unabhängig von der letztlich festgestellten Verletzungsschwere in den jeweiligen Jahren an.

Q3. Wie viele Fälle haben Sie/hat Ihre Klinik in das TraumaRegister der DGU eingeschlossen?

Bitte geben Sie die Anzahl der abgeschlossenen Fälle in den jeweiligen Jahren an.

Dabei ist die Schwere der Fälle/Basiskollektiv für diese Angabe nicht relevant.

Falls nicht zur Hand finden Sie die Zahlen in Ihren Klinik-Jahresberichten.

Diese stehen den Inhabern der Rolle „Klinikadministrator“ des TraumaRegisters im Traumaportal zum Download zur Verfügung.

Falls vorliegend: Q4) Wie viele Fälle konnten wegen fehlender Einwilligungserklärung nicht in das TraumaRegister DGU eingeschlossen werden?

Bitte geben Sie die Anzahl der Fälle in den jeweiligen Jahren an.

Falls vorliegend: Q5. Wie viele Fälle konnten aufgrund anderer bzw. organisatorischer Gründe nicht in das TraumaRegister DGU eingeschlossen werden?

Bitte geben Sie die Anzahl der Fälle an in den jeweiligen Jahren.
